# Mga Modulates Bmpr1a Activity by Antagonizing Bs69 in Zebrafish

**DOI:** 10.3389/fcell.2018.00126

**Published:** 2018-09-28

**Authors:** Xiaoyun Sun, Ji Chen, Yanyong Zhang, Mumingjiang Munisha, Scott Dougan, Yuhua Sun

**Affiliations:** ^1^Institute of Hydrobiology, Chinese Academy of Sciences, Wuhan, China; ^2^Department of Cellular Biology, University of Georgia, Athens, GA, United States

**Keywords:** Mga, Bmp signaling, Zmynd11, Bs69, Bmpr1a, ventral tailfin

## Abstract

MAX giant associated protein (MGA) is a dual transcriptional factor containing both T-box and bHLHzip DNA binding domains. *In vitro* studies have shown that MGA functions as a transcriptional repressor or activator to regulate transcription of promotors containing either E-box or T-box binding sites. BS69 (ZMYND11), a multidomain-containing (i.e., PHD, BROMO, PWWP, and MYND) protein, has been shown to selectively recognizes histone variant H3.3 lysine 36 trimethylation (H3.3K36me3), modulates RNA Polymerase II elongation, and functions as RNA splicing regulator. Mutations in MGA or BS69 have been linked to multiple cancers or neural developmental disorders. Here, by TALEN and CRISPR/Cas9-mediated loss of gene function assays, we show that zebrafish Mga and Bs69 are required to maintain proper Bmp signaling during early embryogenesis. We found that Mga protein localized in the cytoplasm modulates Bmpr1a activity by physical association with Zmynd11/Bs69. The Mynd domain of Bs69 specifically binds the kinase domain of Bmpr1a and interferes with its phosphorylation and activation of Smad1/5/8. Mga acts to antagonize Bs69 and facilitate the Bmp signaling pathway by disrupting the Bs69–Bmpr1a association. Functionally, Bmp signaling under control of Mga and Bs69 is required for properly specifying the ventral tailfin cell fate.

## Introduction

Bone morphogenetic proteins (BMPs) comprise a subgroup of the TGF-beta family of secreted signaling molecules. They transduce their signal by extracellular binding to membrane protein complex consisting of a type I receptor (BMPRI) and a type II receptor (BMPRII). Type I BMP receptor (BMPRI) activation leads to the phosphorylation and activation of Smad1/5/8. The pSmad1/5/8 form complex with SMAD4 and translocate to the nucleus to regulate transcription of downstream target genes. BMP signaling is known to control multiple important biological events, ranging from dorsal-ventral patterning, stem cell maintenance and differentiation to tissue homeostasis (Katagiri and Watabe, 2016). Given its importance in development and homeostasis, Bmp signaling is tightly regulated at the extra- and intracellular levels, by numerous factors such as Noggin, Chordin, Smad7, and Fkbp1A (Wang et al., 2014).MAX’s giant associated protein (MGA) was first identified as a MAX interacting protein by a yeast two hybrid assay in a mouse embryonic day e9.5 and e10.5 cDNA library (Hurlin et al., 1999). Like other Myc family of transcriptional factors, MGA has a basic helix-loop-helix zipper (bHLHZip) domain that mediates dimerization with MAX, which is required for their specific DNA binding to E-box sequences. In addition to the bHLHZip domain, MGA contains a second DNA-binding domain, the T-box or T-domain. *In vitro* studies suggested that MGA could regulate transcription of promotors containing either E-box or T-box binding sites (Hurlin et al., 1999). MGA is thus proposed to function as a dual-specificity transcription factor that could regulate the expression of both the MAX-network and the T-box gene family genes. MGA:MAX heterodimers were often found as part of a large transcription-silencing complex E2F6-com.1 or as part of polycomb repressive complex 1 (PRC1) that catalyzes the monoubiquitylation of histone H2A (Ogawa et al., 2002; Gao et al., 2012). MGA:MAX were shown to repress developmental genes in somatic or embryonic stem cells by recruiting PRC1.6 complex to gene promotors (Endoh et al., 2017; Suzuki et al., 2017). Consistently, MGA depletion leads to the death of proliferating pluripotent ICM cells *in vivo* and *in vitro*, and the loss of self-renewal and pluripotency of embryonic stem cells (Washkowitz et al., 2015). Moreover, Mga mutation in somatic cells is associated with a variety of tumor or cancers, including aggressive lymphoma called Richter’s Syndrome, which occurs in a minority of patients with chronic lymphocytic leukemia (De Paoli et al., 2013; The Cancer Genome Atlas Research Network, 2014; Jo et al., 2016). Together, these studies suggest that MGA functions as a tumor suppressor in normal tissues, presumably by antagonizing Myc oncogene or by recruiting PRC1 to target genes.

Because Mga deficient mice are embryonic lethal, the role of MGA in vertebrate embryogenesis and disease remains unclear. To overcome this issue, we and others took advantage of the zebrafish model system, which is useful for developmental biology studies because of its transparent embryo during early embryogenesis and it is also highly amenable for genetic studies. By Morpholino-mediated gene knockdown, Rikin and Evans (2010) showed that Mga plays essential role in organogenesis by regulating gata4 expression. We recently reported that Mga together with Smad4 and Max are required for the dorsal ventral patterning of zebrafish embryos by transcriptionally regulating Bmp2 expression in the extra embryonic tissue, yolk syncytial layer (YSL) (Sun et al., 2014). This was the first report showing that Mga is involved in the regulation of Bmp signaling in a vertebrate. Unlike other Myc family of transcriptional factors, Mga is predominantly localized in the cytoplasm throughout early zebrafish embryogenesis. This observation implies that Mga has important roles in the cytoplasm, and it may also regulate Bmp signaling independent of its transcriptional activities.

BS69 (ZMYND11) is a multidomain-containing (i.e., PHD, BROMO, PWWP, and MYND) protein that was originally identified as an adenoviral early region 1A-interacting protein (Ansieau and Leutz, 2002; Harter et al., 2016). Through its PHD–BROMO–PWWP domains, BS69 selectively recognizes histone variant H3.3 lysine 36 trimethylation (H3.3K36me3), modulates RNA Polymerase II elongation, and functions as RNA splicing regulator for intron retention (Guo et al., 2014; Wen et al., 2014). The MYND domain of BS69 seems to act as an important protein–protein interaction surface, through which BS69 interacts with a variety of chromatin regulators, including MGA (Velasco et al., 2006). Recently, a growing body of research has shown that BS69 localized in the cytoplasm or cytoplasmic membrane is involved in mediating multiple signaling pathways. For instances, BS69 physically interacts with LMP1 and negatively regulates LMP1-mediated JNK and Nf-κB activation (Hateboer et al., 1995; Chung et al., 2002; Wan et al., 2006; Ikeda et al., 2009). BS69 associates with lymphotoxin beta receptor (LTβR) and inhibits LTβR-mediated signaling transduction (Liu et al., 2011). It has been shown that BRAM1, an alternatively spliced form of BS69, may inhibit Bmp signaling by interacting with the type I BMP receptor 1A (Kurozumi et al., 1998; Morita et al., 2001; Wu et al., 2006). However, BRAM1 was thought likely to be an artificial product that generated from the library construction (Velasco et al., 2006). Therefore, it remains unclear whether BS69 is involved in the regulation of Bmp signaling.

Using zebrafish as a model, we revealed a cytoplasmic role for Mga in the regulation of Bmp signaling. We showed that Mga protein localized in the cytoplasm acts to antagonize Bs69 to facilitate the Bmp signaling pathway. Mechanistically, Mga binds to Bs69 and disrupts the Bs69–Bmpr1a association, thereby maintaining proper Bmp signaling that is required to properly specify zebrafish ventral tail fin.

## Materials and Methods

### Zebrafish Maintenance

Zebrafish (*Danio rerio*) were maintained at 28.5°C on a 12 h light/12 h dark cycle. All procedures were performed with the approval of the Institute of Hydrobiology, Chinese Academy of Sciences, Wuhan, China.

### Generation of *mga* and *bs69* Mutants Using TALEN or CRISPR/Cas9

*Mga* mutants (GenBank accession numbers MH853640-853641) were generated by TALEN technology as described (Chen et al., 2016). We identified potential TALEN-target sites in the coding sequence of zebrafish *mga* gene (NM_001170739.1) using ZiFit 3.0^1^. The *mga* TALEN recognition sequences are: left TALEN 5′-CCATTGCAGCCCAGCCTG-3′ and right TALEN 5′-GAATGAGACGAACAGTT-3′. Between the two binding sites is an 16-bp spacer (GAGGATGTCGAAGGTC). Genotyping was conducted using PCR followed by restriction enzyme digestion. The primers used were 5′-TTCTGACAACAGTATTTCCA-3′ and 5′-CTCGTTCTAAACTCGGTTGACT-3′. *Bs69* mutants (GenBank accession numbers MH853642-853643) were generated by CRISPR/Cas9 technology. gRNA was designed to target a site 5′-GGCTGATGTGGAACAGCTGT-3′ in exon 15 of *bs69* gene. Genotyping was conducted using PCR followed by restriction enzyme digest. The primers were 5′-CCCTTACAGTCTCCTCCTGTAT-3′ and 5′-TGTTCTCCGCCTTCATCATTT-3′. Mutagenized F0 males were crossed to wild-type females to obtain F1 fish. The F1 heterozygous females were then crossed with wild type males to derive the F2 heterozygous. The F2 heterozygous fish were randomly intercrossed, yielding F3-offspring.

### Plasmid Constructions and Microinjections

To make PCS2- version of constructs used in this work, cDNAs encoding zebrafish Mga, Bs69 and their mutants were generated by RT-PCR from cDNA libraries made from 8 hpf zebrafish embryos, and then cloned into the pCS2+ vector using the In-fusion HD Cloning kit (Clontech). The primers used are shown in **Table 1**. *Mga, bs69* and *bmpr1aa* and mutant mRNAs were made using mMESSAGE mMACHINE^®^ Kit (Ambion, TX, United States). mRNAs were injected to the zebrafish embryos at one-cell-stage by a microinjector (WPI, United States).

**Table 1:** Primer used for amplifying the indicated cDNAs.

*Name*	*Forward sequence (5′–3′)*	*Reverse sequence (5′–3′)*
FLAG-Bs69	*ATGGATTACAAGGATGACGACGATAAGTTGGAACTGGCCACGATGTC*	*TACCCAATAGCGTGTCTCGTG*
FLAG-Bs69Δ	*ATGGATTACAAGGATGACGACGATAAGTTGGAACTGGCCACGATGTC*	*TCACCACTGCTTCTTCTTGGTC*
HA-Bs69-Mynd	*ATGTACCCATACGATGTTCCAGATTACGCTGAGCCCGAGATGGAAGCAG*	*TACCCAATAGCGTGTCTCGTG*
Bmpr1a-FLAG	*ACAATGCGTCAGCTTTTGTTCATCAC*	*TCACTTATCGTCGTCATCCTTGTAA TCGATTTTAATGTCTTGAGATTCCAC*
Bmpr1a-kinase-FLAG	*ATGATCGGAAAAGGACGATATGG*	*TCACTTATCGTCGTCATCCTTGTAATC GATTTTAATGTCTTGAGATTCCAC*
FLAG-Mga-Cter	*ATGGATTACAAGGATGACGACGATAAGATGAATCTGCTCGACGTCACACTG*	*TCACATTTGTGGTGTATCTTGCTC*
FLAG-MgaΔ	*ATGGATTACAAGGATGACGACGATAAGATGAATCTGCTCGACGTCACACTG*	*TCAAGGCCGCCATGTCACACTG*

### Cell Culture and Transfection

HEK293 or HEK293T cells were cultured with DMEM (BI) containing 10% fetal bovine serum (FBS, Gibco). Cell transfection was performed with Lipofectamine 2000 (Invitrogen) according to the manufacturer’s instructions. Briefly, cells (about 10^6^ viable cells) were seeded in 6-well plates without antibiotics in DMEM medium containing 10% FBS. Then the constructs expressing the tagged protein of interest or empty pCS2+ vector were transiently transfected into HEK293 cells. 48 h later, the cells were harvested for co-immunoprecipitation and Western blot analysis.

### Luciferase Assay

The BRE-luc reporter was kindly provided by Prof. Zongbin Cui. pTK-Renilla was kindly provided by Dr. Xing Liu (Liu et al., 2015). HEK293 or C2C12 cells (about 10^5^ viable cells) were seeded in 24-well plates in DMEM medium containing 10% FBS for 24 h. The cells were then transiently transfected with indicated luciferase reporters using Lipofectamine 2000 (Invitrogen). pTK-Renilla was used as an internal control. After transfection, the cells were treated with BMP4 (10 ng/ml) or control for 16 h. The luciferase activity was measured with the Dual-luciferase Reporter Assay system (Promega).

### Co-immunoprecipitation and Western Blot Analysis

The protocols for the co-immunoprecipitation and WB analysis were described (Sun et al., 2011). For pSmad1/5/9 WB analysis, the phosphatase inhibitor (Thermo, A32957) was used. For *in vivo* co-immunoprecipitation assay, whole cell lysates were prepared with the TEN buffer (50 mM Tris-HCL, 150 mM NaCl, 5 mM EDTA, 1% Triton X-100, 0.5% Na-deoxycholate, and ROCHE protease inhibitor cocktail). The cytoplasmic and nuclear fractions were prepared with a ProteinExt^®^ Mammalian Nuclear and Cytoplasmic Protein Extraction Kit (TransGen, DE201-01). 1.5 mg Dynabeads protein G was conjugated with 10 μg anti-Mga antibodies, 10 μg anti-Bs69 antibody, 10 μg anti-FLAG antibody, 10 μg anti-HA antibody, or 10 μg IgG. The cell lysates and the antibody-conjugated Dynabeads were incubated overnight at 4°C. After extensive washing, the beads-protein complex were boiled and the supernatant was loaded on a 8% PAGE gel for electrophoresis. The proteins were transferred to a PVDF membrane, followed by blocking with 5% (w/v) non-fat milk for 2-h at room temperature. Then the membrane was incubated overnight at 4°C with the primary antibodies. Anti-MgaN (STTPSENLPADAR); anti-MgaI (EHSADKNTLKSSDQN); anti-MgaC (SPDSKDEIDIPPK) were made by Genscript (China). Anti-FLAG(1B10) and anti-HA (4F6) were purchased from Abbkine (China). Anti-pSmad1/5/9 (D5B10) was purchased from the Cell Signaling Technology. HRP-conjugated goat anti-rabbit IgG (GtxRb-003-E3EUR) was purchased from ImmunoReagents. Anti-mouse IgG, HRP-linked antibody (#7076s) was purchased from Cell Signaling Technology.

### Protein–Protein Interaction Assay Using Rabbit Reticulocyte Lysate System

FLAG or HA tagged Mga, Bs69 or Bmpr1a proteins were synthesized using the TnT coupled reticulocyte lysate system according to the manual (L5020, Promega, United States). Briefly, 1 μg of circular PCS2- version of plasmid were added directly to the TnT Lysate and incubated in a 50 μl reaction for 1.5 h at 30°C. To evaluate the quality or quantity of the synthesized protein, 1 μl of the reaction products were subjected to WB assay.

For protein–protein interaction assay, 5–10 μl of the synthesized HA or FLAG tagged proteins were mixed in a 1.5 ml tube with the TEN buffer, and the mixture was shaken for 30 min at room temperature. Next, Co-IP or pull-down assay was performed using Dynabeads protein G coupled with FLAG or HA antibodies as described above (Sun et al., 2014).

### Immunofluorescence Assay

Six to eight hpf zebrafish embryos were collected and fixed with 4% paraformaldehyde (PFA) overnight at 4°C. Embryos were washed with PBST (0.1% Triton X-100) and then permeabilized with acetone for 7 min at -20°C. The embryos were rinsed with PBST, followed by 1 h blocking with solution (0.1% Triton X-100, 1% BSA, and 1% DMSO in PBS). Then the embryos were incubated with primary antibodies overnight rocking at 4°C. After washing with the blocking solution, embryos were incubated with the secondary antibodies for 2 h at room temperature, followed by extensively washing. Nuclei was stained with DAPI. Primary antibodies diluted with blocking solution were anti-pSmad1/5/9 (1:300), anti-FLAG (1:300), and anti-HA (1:300). Secondary antibodies diluted with blocking solution were goat anti-mouse IgG(H+L) Alexa Fluor 555 (1:500, Molecular Probes), goat anti-rabbit IgG(H+L) Alexa Fluor 488 (1:500, Molecular Probes). The embryos were transferred to a Glass Bottom (NEST) and submerged with 75% glycerol. The images were taken under a Leica SP8 confocal microscope (Germany).

### Whole-Mount *in situ* Hybridization

Whole-mount *in situ* hybridization (WISH) was performed as described previously (Sun et al., 2011). The DIG-labeled probes were generated with DIG RNA Labeling Kit (SP6/T7) (Roche) and Fluorescein-labeled RNA probes were made with Fluorescein RNA Labeling Mix, 10x conc. (Roche). *Mga, chordin*, and *eve1* RNA *in situ* probes were described before (Sun et al., 2014). Zebrafish embryos were collected at different development stages and fixed with 4% PFA overnight at 4°C. Following the WISH, the embryos were transferred to 6-well plates and submerged by 100% glycerol for imaging.

## Results

### Mga Positively Regulates Bmp Signaling

**FIGURE 1:**
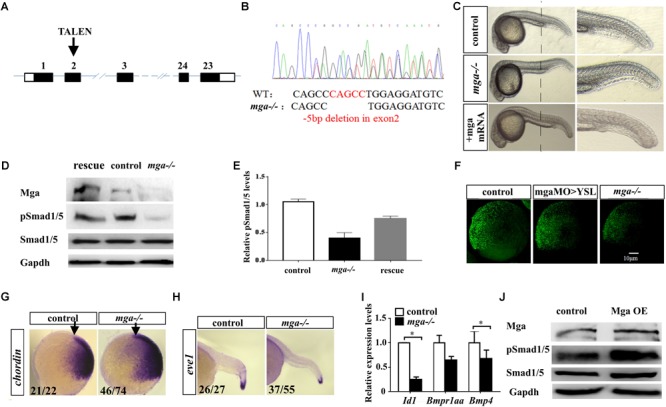
Mga mutant embryos exhibited mild dorsalized phenotype. **(A)** Schematic representation of the zebrafish *mga* gene, depicting the location of the TALEN targeting site. **(B)** Sequences around the TALEN targeting site, showing the TALEN-induced 5-bp deletion in *mga* (in red). **(C)** Phenotypes of 1 dpf wild type, *mga* mutant, and *mga* mutant embryos injected with 50 pg *mga* mRNA at one-cell-stage. The reduction of ventral tail fin was restored by injecting 50 pg *mga* mRNA into one-cell-stage of mga mutant embryos. Lateral view. **(D)** Immunoblot analysis of Mga and pSmad1/5 levels of lysates from 7 hpf control, mutant and *mga* mRNA restored embryos. **(E)** Quantification of pSmad1/5 levels of panel D based on three independent experiments. **(F)** pSmad1/5 gradient of wild type, mga > YSL morphants, and *mga* mutant embryos at 7 hpf. Dorsal to the right. **(G)** *Chordin* expression in *mga* mutant and control embryos at shield stage. Lateral view, and dorsal to the right. **(H)** *eve1* expression in *mga* mutant and control embryos at 22 hpf. Lateral view, and dorsal to the right. **(I)** qRT-PCR transcript analysis of the indicated Bmp target genes in control and *mga* mutant embryos at 8 hpf. **(J)** Immunoblot analysis of pSmad1/5 levels of lysates from 8 hpf control and Mga overexpressing (OE) embryos. All experiments were performed in technical triplicate and are representative of multiple experiments. ^∗^*p* < 0.05.

To establish a genetic model to explore the developmental function of Mga, and avoid potential off-target effects of Morpholino Oligos, we generated *mga* mutant zebrafish by using TALEN technology (**Figure 1A**). Out of 12 potential founders, we identified two fish in which *mga* gene was mutated at the TALEN cleavage site in the exon 2. The identity of each mutation was confirmed by genotyping (**Figure 1B**). Each mutation results in an open reading frame-shift that leads to a premature stop codon. Western blotting was used to detect Mga protein in lysates from homozygous mutant and control embryos at 8 hpf, using Mga specific antibodies (Sun et al., 2014). As seen in **Figure 1D**, the band around 250 kDa was detected in lysates from control embryos, but was barely detected in lysates from mutant embryos. These results indicate that our mutant alleles are functional nulls. One mutation line with a 5 bp deletion in exon 2 of the *mga* gene, was used for most of the subsequent studies (**Figure 1B**).

The mutant embryos at 1 dpf appear largely normal except the loss or reduction of the ventral tail fin defect (**Figure 1C**). Because *mga* mutant embryos at 1 dpf exhibited the loss of ventral tailfin defect that resembled our previously characterized *mga* > YSL morphants, we went on to confirm that Bmp signaling was compromised in *mga* mutant embryos (Sun et al., 2014). Nuclear phosphorylated Smad1/5/8 is a direct intracellular readout of Bmp signaling. As expected, pSmad1/5 levels were indeed reduced in mutant embryos. Importantly, pSmad1/5 levels could be rescued by injecting 100 pg *mga* mRNA, demonstrating the specificity of observed phenotype (**Figures 1D,E**). A low but detectable level of phospho-Smad1/5 was still present in lysates of mutant embryos, suggesting that Mga is only required for higher Bmp activity. This was in accordance with the notion that the ventral tail fin formation is most sensitive to the reduction of Bmp signaling (Schumacher et al., 2011). We also compared the pSmad1/5 gradient among 7 hpf *mga* > YSL morphants, *mga* mutant and control embryos by immunofluorescence assay using anti-pSmad1/5/9 antibody. As seen from **Figure 1F**, *mga* mutant embryos and *mga* > YSL morphants both exhibited reduced pSmad1/5 gradient when compared with the control embryos. However, the pSmad1/5 gradient in *mga* mutant embryos was reduced to a greater extent than *mga* > YSL morphants (**Figure 1F**), suggesting that Mga cell autonomously regulates Bmp signaling in embryos in addition to its role in YSL (Sun et al., 2014). To further confirm that Bmp activity was reduced in *mga* mutants, whole mount *in situ* hybridization was performed to examine the expression pattern of dorsal marker *chordin* and the ventral marker *eve1*. As expected, *chordin* expression was slightly expanded in mutant embryos at shield stage, whereas *eve1* expression was slightly reduced in mutant embryos at 22 hpf compared with the controls (**Figures 1G,H**). Moreover, the expression of well-known Smad-dependent Bmp target genes *Id1* and *bmp4* was down-regulated in *mga* mutant embryos (**Figure 1I**).

On the other hand, we overexpressed Mga by injecting 200 pg *mga* mRNA into one-cell-stage wild-type embryos. Beta-gal overexpressing embryos were used as controls. As seen in **Figure 1J**, pSmad1/5 levels were slightly elevated in Mga overexpressing embryos compared with control embryos, which further supports that Mga positively regulates Bmp signaling.

Taken together, we concluded that Mga is cell autonomously required for proper Bmp signaling that is important for specifying the ventral tailfin cell fate during zebrafish embryogenesis.

### Mga Interacts With Bs69 in Zebrafish Embryos

Our yeast two hybrid experiments have identified multiple Mga interacting proteins, including Smad1, Smad4, and type I Bmp receptors, suggesting that Mga could regulate Bmp signaling by physical association with the core components of Bmp signaling pathway (Sun et al., 2014). Type I Bmp receptor 1a (Bmpr1a) was of particular interest to us because previous studies have shown that *bmpr1a* mutant embryos at 1 dpf exhibited the loss or reduction of ventral tailfin defect that closely resembled our *mga* mutant embryos (Smith et al., 2011), and also that wild type embryos injected with mRNA encoding dominant negative Bmpr1a (dnBmpr1a) at one-cell-stage had defects in the ventral tailfin formation at 1 dpf (Pyati et al., 2005) (**Supplementary Figure S1A**). Wild type embryos treated with 0.05 μM dorsomorphin or LDN193189, which are known potent Bmpr1a inhibitors, exhibited the loss or reduction of ventral tail fin defect at 1 dpf (**Supplementary Figure S1A**). Furthermore and most importantly, injection lower dose (25 pg) constitutive active *bmpr1a* (*caBmpr1a*) mRNA into one-cell-stage *mga* mutant embryos rescued the loss or reduction of the ventral tailfin phenotype (Nikaido et al., 1999) (**Supplementary Figure S1B**). Altogether these previous studies, along with our observations, strongly suggested a functional link between Mga and Bmpr1a. Unfortunately, we failed to detect reproducible interaction between Mga and Bmpr1a (data not shown). Transcriptional regulation of *bmpr1a* gene by Mga was ruled out, as *bmpr1a* transcript levels in *mga* mutant embryos were comparable to control embryos (**Figure 1I**).

It has been previously shown that mammalian MGA directly interacts with BS69, and that BRAM1, a possible spliced form of BS69, may be involved in the regulation of BMPR1A activity (Kurozumi et al., 1998; Ansieau and Leutz, 2002; Velasco et al., 2006; Wu et al., 2006). Therefore, we speculated that Mga may modulate Bmpr1a activity through Bs69 in zebrafish. To test these hypothesis, we firstly examined the gene expression pattern of *mga, bs69*, and *bmpr1a* during zebrafish early embryogenesis. The three genes have similar expression patterns from blastula to late organogenesis as reported (Wu et al., 2006; Rikin and Evans, 2010; Smith et al., 2011). During gastrulation, both *bmpr1aa* and *mga* transcription levels are high, whereas *bs69* transcript levels are relatively low. During early somitogenesis stage, *mga* and *bmpr1aa* are strongly expressed in the trunk and tail region, whereas *bs69* expression domain seems to be more restricted to the ventral region of the trunk. During organogenesis, all three genes are strongly expressed in the head and gut regions (**Supplementary Figure S1C**). The contrasting expression patterns between *mga* and *bs69* genes imply that Mga may act to antagonize Bs69 to modulate Bmpr1a activity.

**FIGURE 2:**
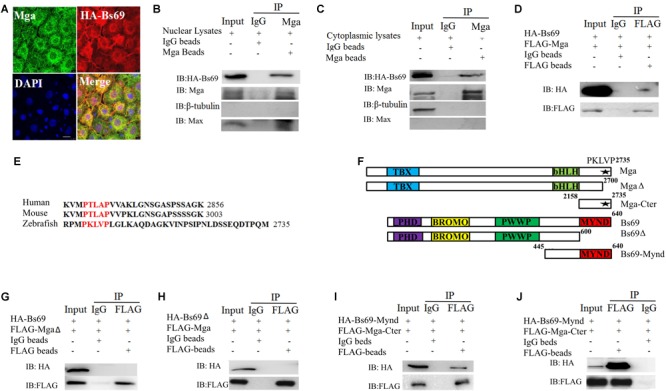
Mga interacts with Bs69. **(A)** Confocal images of Mga and HA-Bs69 proteins in 7 hpf embryos. Scale bar 10 μm. **(B)** Co-immunoprecipitation of Mga and HA-Bs69 in nuclear lysates from 8 hpf embryos. **(C)** Co-immunoprecipitation of Mga and HA-Bs69 in cytoplasmic lysates from 8 hpf embryos. **(D)** Co-immunoprecipitation of HA-Bs69 and FLAG-Mga in 293T cells. **(E)** Identification of a conserved PXLXP motif in human, mouse and zebrafish MGAs at the end of the C-terminal region. The PXLXP conserved motif were highlighted in red. **(F)** Schematic representation of the full-length and truncated Mga and Bs69 constructs as indicated. **(G)** FLAG-MgaΔ did not immunoprecipitate with HA-Bs69 in 293T cells. **(H)** FLAG-Mga did not immunoprecipitate with HA-Bs69Δ in 293T cells. **(I)** FLAG-Mga-Cter immunoprecipitated with HA-Bs69-Mynd in 293T cells. **(J)** Co-immunoprecipitation assay of the *in vitro* translated FLAG-Mga-Cter and HA-Bs69-Mynd.

To investigate whether Mga regulates Bmpr1a activity through Bs69, we determined whether Mga associates with Bs69 in physiological conditions in zebrafish embryos. The cellular co-localization of Bs69 and Mga was examined in 7 hpf embryos (**Figure 2A** and **Supplementary Figure S2A**). One-cell-stage wild-type embryos were injected with 100 pg HA-tagged *bs69* mRNA, and embryos were collected for co-immunofluorescence assay using Mga and HA antibodies. Our IF data clearly showed that these two proteins were co-localized in both cytoplasm and nucleus. To determine whether Mga interacts with Bs69 in physiological conditions, we performed co-immunoprecipitation (co-IP) assay using Mga and HA antibodies. Cytoplasmic and nuclear fractions of lysates from 7 hpf embryos were prepared using a commercial TransGen kit, and were subjected to co-IP experiments. It was obvious that Mga interacts with HA-Bs69 in both cytoplasm and nucleus (**Figures 2B,C**).

Next, we mapped the interacting domain between Mga and Bs69 in 293T cells. We first confirmed that FLAG-Mga and HA-Bs69 interact with each other (**Figure 2D**). It was previously reported that the PXLXP motif of mammalian MGA binds to the MYND domain of BS69 (Ansieau and Leutz, 2002). We identified a conserved PXLXP motif (PKLVP) within the C-terminal region (amino acid 2702-2706) of zebrafish Mga protein (**Figure 2E**). Deletion of this PXLXP motif (FLAG-MgaΔ) abrogated Mga binding to HA-Bs69 (**Figure 2G**). Truncated Bs69 lacking the Mynd domain (HA-Bs69Δ) did not co-immunoprecipitate with the FLAG-tagged Mga (**Figure 2H**). However, FLAG-tagged C-terminal fragment of Mga (FLAG-Mga-Cter) containing the PXLXP motif was sufficient to bind the Mynd domain of Bs69 (HA-Bs69-Mynd) (**Figure 2I**). To determine whether this interaction was direct or not, we used a reticulate lysate system to synthesize FLAG-Mga-Cter and HA-Bs69-Mynd. When they were mixed together, the anti-FLAG antibody readily pull-downed HA-Bs69-Mynd (**Figure 2J**).

Taken together, we concluded that zebrafish Mga physically associates with Bs69 in the physiological condition and this interaction is mediated by the PXLXP motif of Mga and the Mynd domain of Bs69.

### Bs69 Negatively Regulates Bmp Signaling

**FIGURE 3:**
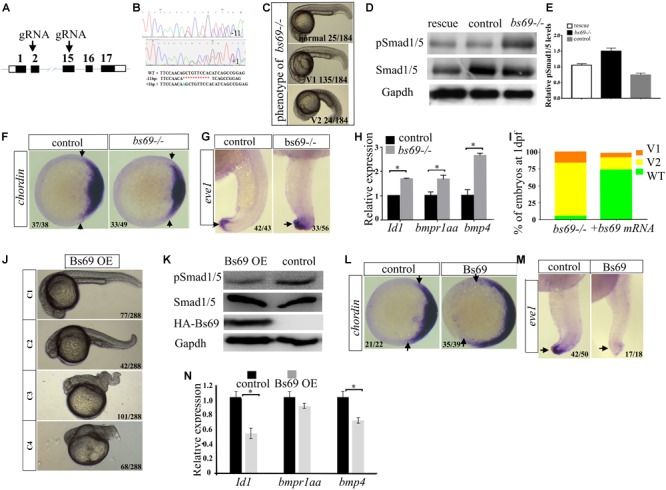
Bs69 negatively regulates Bmp signaling. **(A)** Schematic representation of *bs69* gene, showing the CRISPR/Cas9 targeting site in exon 15. **(B)** Sequences of the two *bs69* mutants at the CRISPR targeting site. CRISPR-induced 11 bp deletion is highlighted in red; CRISPR-induced 1 bp insertion is highlighted in green. **(C)** The phenotypes of *bs69* mutant embryos at 1 dpf. V1: V1 ventralized phenotype; and V2: V2 ventralized phenotype, according to the DV patterning index (Kishimoto et al., 1997). **(D)** Immunoblot analysis of pSmad1/5 levels in lysates from 8 hpf control, *bs69* mutant, and Bs69 restored embryos. **(E)** Quantification of pSmad1/5 levels from panel D. **(F)** *Chordin* expression in *bs69* mutant and control embryos at shield stage. Animal view, and dorsal to the right. **(G)** *eve1* expression in *bs69* mutant and control embryos at 22 hpf. lateral view, and dorsal to the right. **(H)** qRT-PCR analysis of the Bmp target genes in 8 hpf *bs69* mutant and control embryos. **(I)** One-cell-stage *bs69* mutant embryos were injected with 50 pg bs69 mRNA, and the injected embryo at 30 hpf were scored and phenotyped according to the dorsal-ventral patterning index. **(J)** The phenotypes of Bs69 overexpressing embryos at 2 dpf. C1-4 dorsalized phenotypes according to DV patterning index. **(K)** Immunoblot analysis of pSmad1/5 levels of lysates from 8 hpf control and Bs69 overexpressing embryos. **(L)** *Chordin* expression in Bs69 overexpressing embryos at shield stage. Animal view, and dorsal to the right. **(M)** *eve1* expression in BS69 overexpressing embryos at 22 hpf. Lateral view, and dorsal to the right. **(N)** qRT-PCR transcript analysis of the indicated Bmp target genes in 8 hpf control and Bs69 overexpressing embryos. All experiments were performed in technical triplicate and are representative of multiple experiments. ^∗^*p* < 0.05.

To understand the function of Bs69, we generated *bs69* mutant zebrafish by CRISPR/Cas9 technology (**Figure 3A**). We designed gRNAs targeting exon 2 or 15 of bs69. Out of eight potential founders, we identified two fish in which *bs69* gene was mutated around the CRISPR targeting site of exon 2. Both mutations result in an open reading frame-shift that leads to a premature stop codon. Out of nine potential founders, we identified two fish in which *bs69* gene was mutated around the CRISPR targeting site of exon 15. Both mutations result in an open reading frame-shift that leads to a truncated Bs69 protein that lack the entire Mynd domain. One mutant causes 1-bp insertion within the CRISPR targeting site, resulting in a ∼475aa truncated protein. Another mutant has 11-bp deletion within the CRISPR targeting site, resulting in a ∼480aa truncated BS69 protein (**Figure 3B**). The F3 zygotic *bs69* mutants are viable and can be raised up to adulthood. When F3 female and male adults were intercrossed, maternal zygotic *bs69* mutant embryos were obtained for further analysis.

The majority of *bs69* mutant embryos at 1 dpf exhibited mild V1 ventralized phenotype, characterized by slightly reduced head region. Some of the *bs69* mutant embryos at 1 dpf displayed missing notochord, and enlarged ventral cell types, indicating a V2 ventralized phenotype (Kishimoto et al., 1997) (**Figure 3C**). Smad1/5 phosphorylation was increased in 8 hpf *bs69* mutants compared with controls, and this could be rescued by injecting *bs69* mRNA into one-cell-stage mutant embryos (**Figures 3D,E**). To confirm that *bs69* mutants had the ventralized phenotype, whole mount *in situ* hybridization was performed to examine the expression pattern of dorsal marker *chordin* and the ventral marker *eve1*. As expected, the *chordin* expression domain was decreased in mutant embryos at shield stage, while *eve1* expression domain was expanded in mutant embryos at 22 hpf compared with control embryos (**Figures 3F,G**). Moreover, the expression of the known Bmp target genes *Id1* and *bmp4* was examined in *bs69* mutant and control embryos. As seen in **Figure 3H**, the expression of these genes was up-regulated in *bs69* mutant embryos. Injection of 50 pg *bs69* mRNA into one-cell-stage *bs69* mutant embryos largely rescued the ventralized phenotypes (**Figure 3I**).

Next, we overexpressed Bs69 by injecting *bs69* mRNA into one-cell-stage wild-type embryos. Beta-gal overexpressing embryos were used as controls. The Bs69 overexpressing embryos at 24 hpf exhibited dorsalized phenotypes ranging from C1 to C4 dorsalization depending on the injected mRNA dose (**Figure 3J**). Smad1/5 phosphorylation was decreased in Bs69 overexpressing embryos (**Figure 3K**). Importantly, the DV patterning phenotype of Bs69 overexpressing embryos was similar to those of *bmpr1a* mutants or dnBmpr1a overexpressing embryos (Smith et al., 2011). To further confirm that Bs69 overexpressing embryos had dorsalized phenotypes, whole mount *in situ* hybridization was performed to examine the expression pattern of dorsal marker *chordin* and the ventral marker *eve1*. As expected, *chordin* expression domain was expanded in Bs69 overexpressing embryos at shield stage, while *eve1* expression domain was reduced in Bs69 overexpressing embryos at 22 hpf, compared to controls (**Figures 3L,M**). Moreover, the expression of *Id1* and *bmp4* genes was down-regulated in Bs69 overexpressing embryos, which was similar to that of *mga* mutant embryos (**Figure 3N**). Taken together, we concluded that Bs69 is required for the dorsal ventral patterning of zebrafish embryos, and functions as a negative regulator of Bmp signaling.

### Bs69 Regulates Bmp Signaling by Association With Bmpr1a

**FIGURE 4:**
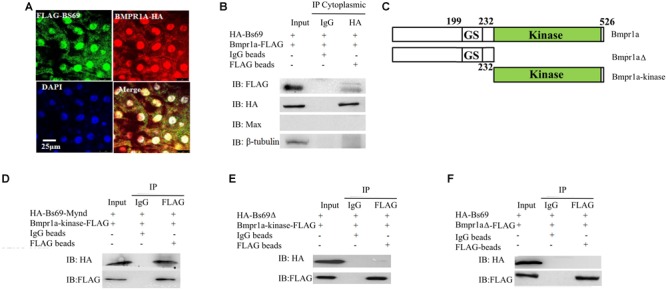
Bs69 associates with Bmpr1a in zebrafish embryos. **(A)** Cellular co-localization of FLAG-Bs69 and Bmpr1a-HA in 7 hpf zebrafish embryos. Scale bar 25 μm. **(B)** Co-immunoprecipitation of HA-Bs69 and Bmpr1a-FLAG in cytoplasmic lysates from 7 hpf embryos. **(C)** Schematic representation of the full-length and truncated Bmpr1a as indicated. **(D)** Co-immunoprecipitation of HA-Bs69-Mynd and Bmpr1a-kinase-FLAG in 293T cells. **(E)** Bmpr1a-kinase-FLAG did not immunoprecipitate with HA-Bs69Δ in 293T cells. **(F)** Bmpr1aΔ-FLAG did not immunoprecipitate with HA-Bs69 in 293T cells.

Next, we investigated the mechanism by which Bs69 negatively regulates Bmp signaling. We hypothesized that Bs69 may regulate Bmp signaling through Bmpr1a in zebrafish. We therefore examined whether Bs69 interacts with Bmpr1a *in vivo*. We injected mRNAs encoding HA-Bs69 and Bmpr1a-FLAG into one-cell-stage *bs69* mutant or wild type embryos, and performed co-immunofluorescence and co-immunoprecipitation assays for 7 hpf embryos. Co-immunofluorescence data clearly showed that HA-Bs69 was co-localized with Bmpr1a-FLAG, and co-immunoprecipitation data demonstrated that HA-Bs69 interacts with Bmpr1a-FLAG (**Figure 4A**). Importantly, HA-Bs69 interacts with Bmpr1a-FLAG in the cytoplasmic fraction of embryonic lysate (**Figure 4B**).

Next, we mapped the interacting domain between Bs69 and Bmpr1a. pCS2-HA-Bs69 or pCS2-HA-Bs69-Mynd and PCS2-Bmpr1a-kinase-FLAG were transiently co-transfected into 293T cells, and co-IP experiments were performed using HA and FLAG antibodies **Figure 4C**. The Mynd domain of Bs69 was sufficient to interact with the kinase domain of Bmpr1a (**Figure 4D**). In contrast, Bs69Δ lacking the Mynd domain did not immunoprecipitate with Bmpr1a, and Bmpr1aΔ lacking the kinase domain did not immunoprecipitate with Bs69 (**Figures 4E,F**).

The association of Bs69 and Bmpr1a in physiological conditions strongly suggested that Bs69 modulates Bmpr1a activity. Like its mammalian counterpart, zebrafish Bs69 also has these three conserved chromatin reader domains (**Figure 2F**). It is possible that zebrafish Bs69 regulates Bmp signaling by modulating chromatin or functioning as a transcriptional co-factor. To investigate the significance of Bs69–Bmpr1a association for the regulation of Bmp signaling, we took advantage of the truncated form of Bs69 (BS69-Mynd) lacking all the chromatin reader domains but containing the intact Mynd domain that was still able to interact with Bmpr1a. By overexpressing Bs69-Mynd or HA-Bs69Δ, we were able to determine whether Bs69 regulates Bmp signaling by physical association with Bmpr1a. To this end, we injected 100 pg mRNAs encoding HA-tagged Bs69-Mynd or HA-Bs69Δ into one-cell-stage wild-type embryos, and collected embryos at 7 hpf or 24 hpf for subsequent assays. HA-Bs69-Mynd was localized in both nuclei and cytoplasm which was similar to HA-Bs69 (**Supplementary Figures S2A–C**). HA-Bs69-Mynd overexpressing embryos displayed dorsalized phenotypes that were similar to HA-Bs69 overexpressing embryos at 1 dpf (**Supplementary Figures S3A,B**). In contrast, HA-Bs69Δ overexpression did not cause obvious dorsalization of the embryos (data not shown). Next, we investigated whether Bs69-Mynd could rescue the DV patterning phenotype of *bs69* mutant embryos. Injection of 50 pg *bs69-mynd* or *bs69* mRNA into one-cell-stage *bs69* mutant embryos largely rescued the ventralized phenotypes at 1 dpf (**Supplementary Figure S3C**). In contrast, injection of 50 pg *bs69Δ* mRNA into one-cell-stage *bs69* mutant embryos had no effect on the DV patterning.

Phosphorylation of Smad1/5/8 at the C-terminal SXS motif by Bmp type I receptor is one of the most critical events in the transduction of Bmp signaling. We hypothesized that Bs69 may negatively regulate Bmp signaling through suppressing Bmpr1a activity by interfering its phosphorylation and activation of Smad1/5. If this is the case, loss of Bs69 function should cause increased Bmp signaling, indicated by elevated pSmad1/5 activity; whereas overexpressing Bs69 should cause decreased Bmp signaling, indicated by diminished pSmad1/5 activity. Indeed, Western blot analyses of Smad1/5 phosphorylation indeed supported this hypothesis (**Figures 3D,J**). Overexpressing HA-Bs69Δ had no obvious effect on the Smad1/5 phosphorylation (data not shown).

Taken together, we concluded that Bs69 negatively regulates Bmp signaling by physical association with Bmpr1a, which interferes with its phosphorylation and activation of Smad1/5.

### Mga Binding to Bs69 Disrupts the Bs69–Bmpr1a Interaction

**FIGURE 5:**
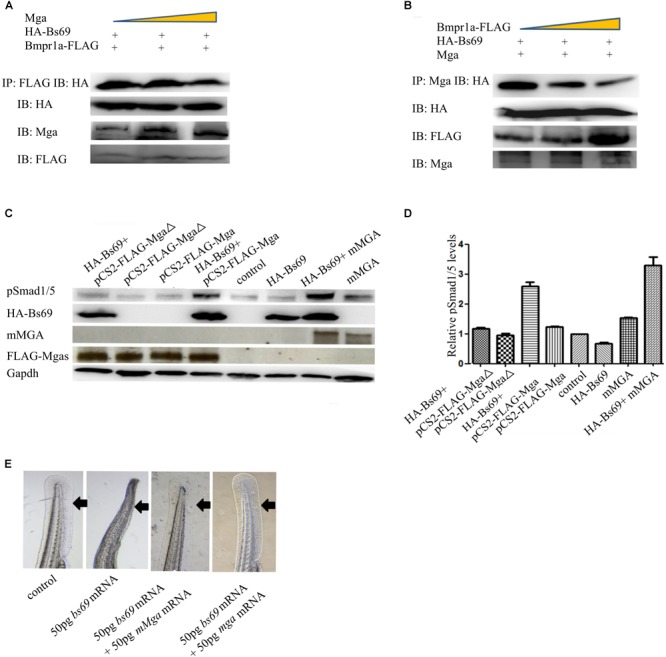
Mga binds to Bs69 and disrupts the Bs69–Bmpr1a interaction. **(A)** Increasing dose of pCS2-Mga (1, 2, 3 μg) along with 1 μg pCS2-HA-Bs69, 1 μg pCS2-Bmpr1a-FLAG were transiently co-transfected into 293T cells, and co-immunoprecipitation of HA-Bs69 and Bmpr1a-FLAG was analyzed. **(B)** Increasing dose of pCS2-Bmpr1a-FLAG (1, 2, 3 μg) along with 1 μg pCS2-HA-Bs69, 1 μg pCS2-Mga were transiently co-transfected into 293T cells, and co-immunoprecipitation of HA-Bs69 and Mga was analyzed. **(C)** One-cell-stage wild-type embryos injected with 50 pg mRNA encoding HA-Bs69 or mixture of mRNAs encoding both HA-Bs69 and mouse MGA, or co-injected with 50 pg mRNA encoding HA-Bs69 with 100ng pCS2-FLAG-Mga or pCS2-FLAG-MgaΔ. Immunoblot analysis of pSmad1/5 levels was performed with lysates from the injected embryos at 8 hpf. **(D)** Relative quantification of pSmad1/5 levels of panel C. **(E)** One-cell-stage wild-type embryos injected with 50 pg mRNA encoding HA-Bs69 or mixture of mRNAs encoding both HA-Bs69 and mouse or zebrafish MGAs, and the ventral tail fin phenotypes of these embryos at 48 hpf were shown.

Because both Mga and Bmpr1a interact with Bs69 through its Mynd domain, we hypothesized that Mga modulates Bmpr1a activity through Bs69. To explore this, we examined the relationship between Mga–Bs69 and Bmpr1a–Bs69 interactions. We performed competitive protein-binding assay. The amount of HA-Bs69 co-immunoprecipitated with Bmpr1a-FLAG became reduced by the increased addition of Mga (**Figure 5A**), and the amount of HA-Bs69 co-immunoprecipitated with Mga became reduced by the increased addition of Bmpr1a-FLAG (**Figure 5B**). These data suggested that Mga and Bmpr1a compete for the binding to Bs69.

Since Mga and Bmpr1a compete for the binding to Bs69, we speculated that Mga functions to maintain or enhance Bmp signaling by antagonizing Bs69 in physiological conditions. We injected into one-cell-stage wild-type embryos with 50 pg mRNA encoding HA-Bs69 or mixture of mRNAs encoding both HA-Bs69 and mouse or zebrafish MGAs. Embryos injected with 50 pg *beta-gal* mRNA were used for controls. Phosphorylation of Smad1/5 was detected by Western immunoblotting of lysates from 8 hpf embryos. As seen in **Figure 5C**, the pSmad1/5 levels in HA-Bs69 overexpressing embryos were reduced compared with control embryos. The pSmad1/5 levels were restored and even enhanced by simultaneously expressing either zebrafish or mouse MGAs, but not by MgaΔ that is unable to interact with Bs69 (**Figures 5C,D**). Accordingly, zebrafish or mouse MGAs rescued the loss of ventral tailfin phenotype in Bs69 overexpressing embryos at 1 dpf, supporting that Bmp signaling under control of Mga and bs69 is required for specifying the ventral tailfin cell fate (**Figure 5E**).

**FIGURE 6:**
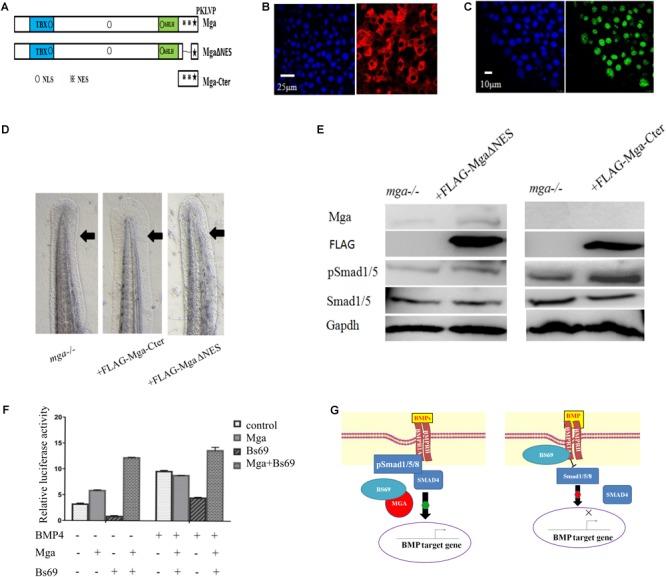
Mga localized in the cytoplasm regulates Bmp signaling. **(A)** Diagram showing the location of putative NLS and ENS in Mga protein. ■: NLS, ■: NES. **(B)** IF showing the cytoplasmic localization of FLAG-Mga-Cter in 7 hpf embryos. **(C)** IF showing the nuclear localization of FLAG-MgaΔNES in 7 hpf embryos. **(D)** FLAG-Mga-Cter but not FLAG-MgaΔNES rescued the reduced ventral tailfin phenotype of *mga* mutant embryos at 2 dpf. **(E)** pSmad1/5 levels were increased in 7 hpf *mga* mutant embryos injected with 100 pg FLAG-Mga-Cter but not FLAG-MgaΔNES. **(F)** The BRE-luc activity assay for Mga and Bs69 in C2C12 cells. **(G)** Cartoon model of how Mga regulates Bmp signaling. Left: Mga localized in the cytoplasm associates with Bs69 which allow Bmpr1a to phosphorylate and activate Smad1/5/8. The pSmad1/5/8 form complex with Smad4 and translocate into nuclei to regulate Bmp target gene expression. Right: In the absence of Mga, Bs69 associates with Bmpr1a and interferes with its phosphorylation and activation of Smad1/5/8 which caused reduced Bmp signaling. All experiments were performed in technical triplicate and are representative of multiple experiments.

That Mga co-localizes and interacts with Bs69 in the cytoplasm strongly suggested that Mga localized in the cytoplasm functions to maintain Bmp signaling through Bs69–Bmpr1a axis. To test this hypothesis, we went on to generate completely nuclear or cytoplasmic version of Mga mutants. Using the NetNES or NLStradamus servers (la Cour et al., 2004; Nguyen et al., 2009), three putative nuclear localization sequences (NLSs) and two putative nuclear export sequences (NESs) were identified for Mga (**Figure 6A**). We injected mRNA encoding FLAG-MgaΔNES or FLAG-Mga-Cter into one-cell stage *mga* mutant zebrafish embryos, and collected 7 hpf embryos for assays described below. Our immunofluorescence assay showed that FLAG-MgaΔNES was strictly localized in the nuclei, whereas FLAG-Mga-Cter was found to be localized only in the cytoplasm (**Figures 6B,C**). These data indicated that we have successfully generated nuclear or cytosolic version of Mga protein. If it was Mga in the cytoplasm that regulates Bmp signaling through Bs69–Bmpr1a axis, then only cytosolic but not nuclear version of Mga protein could rescue the DV patterning defect of *mga* mutant. Indeed, we found that FLAG-Mga-Cter but not FLAG-MgaΔNES rescued the reduced ventral tailfin phenotype of 2 dpf *mga* mutant embryos (**Figure 6D**). Consistently, Smad1/5 phosphorylation was increased in 7 hpf *mga* mutant embryos by overexpressing FLAG-Mga-Cter but not by FLAG-MgaΔNES (**Figure 6E**). Finally, FLAG-Mga-Cter but not FLAG-MgaΔNES rescued the loss of ventral tailfin phenotype in Bs69 overexpressing embryos (data not shown). Together, these data strongly indicated that the role of Mga in the regulation Bmp signaling is mainly acting in the cytoplasm separately from its role as a DNA binding protein.

To further determine how Mga–Bs69 interaction affects Bmp signaling, luciferase activity assays were performed with a Bmp-responsive luciferase reporter BRE-luc. C2C12 cells were co-transfected overnight with pCS2-Mga, PCS2-Bs69, and BRE-luc, followed by 12 h serum starvation, and treated with BMP4 or left untreated for 16 h. As seen from **Figure 6F**, BMP4 treatment remarkably stimulated the Bmp-responsive BRE-luc activity and Bs69 inhibited it. When Mga and Bs69 were co-expressed, Mga substantially antagonized the inhibitory effect of Bs69 on the luciferase activity.

If Mga regulates Bmp signaling through Bs69–Bmpr1a axis, embryos simultaneously depleted of both Mga and Bs69 should have similar DV patterning phenotype to bs69 mutant embryos. To test this hypotheses, we depleted Mga by injecting 4–5 ng *mga* morpholino (mgaMO) into one-cell stage of *bs69* mutant embryos. 4–5 ng mgaMO was shown to cause a reduction of ventral tailfin phenotype in wild-type embryos (Sun et al., 2014). We found that mgaMO had no obvious effect on DV patterning phenotype of 1 dpf *bs69* mutants (**Supplementary Figure S4**).

Together, our data suggested that Mga antagonizes Bs69 to enhance the phosphorylation and activation of Smad1/5/8 both *in vitro* and *in vivo*.

Previous work showed that there are cardiac laterality defects in *bmpr1a* mutant embryos (Smith et al., 2011). If Mga regulates Bmp signaling through Bs69–Bmpr1a axis, *mga* mutant or Bs69 overexpressing embryos should exhibit similar laterality phenotypes as *bmpr1a* mutant embryos. We therefore investigated whether *mga* mutant or Bs69 overexpressing embryos had the cardiac laterality defects. Whole mount *in situ* hybridization was performed to examine the expression of a set of laterality genes, including *spaw, lefty2* and *cmcl2*. Embryos treated with 0.05 μM dorsomorphin or LDN193189 were used as positive controls. A small percentage of *mga* mutant or Bs69 overexpressing embryos indeed displayed the left-right patterning defects similar to *bmpr1a* mutants or dorsomorphin treated embryos (**Supplementary Figures S5A,B**) (Smith et al., 2011). Importantly, Mga or Mga-Cter but not MgaΔ partially rescued the laterality defects of *mga* mutant or Bs69 overexpressing embryos (**Supplementary Figure S5C**).

## Discussion

In this work, we demonstrated that Mga protein localized in the cytoplasm regulates Bmp signaling at least partly by physically interacting with and antagonizing Bs69. We provided genetic and biochemical evidence that Bs69 is a negative regulator of Bmp signaling. The Mynd domain of Bs69 binds to the kinase domain of Bmpr1a which interferes with its phosphorylation and activation of Smad1/5. Mga binds to Bs69 and disrupts the Bs69–Bmpr1a interaction which allows Smad1/5 to be phosphorylated, and proper Bmp signaling to be maintained. Functionally, the Bmp signaling under control of Mga is important for specifying the ventral tailfin cell fate in zebrafish embryos (**Figure 6G**).

### Bs69 Functions as a Negative Regulator of Bmp Signaling by Association With Bmpr1a

BRAM1, previously thought as an alternatively spliced product of BS69, was first identified by a yeast two hybrid screen using BMPR1A as a bait in human cells (Kurozumi et al., 1998). However, based on the analysis of genomic sequences and *Bs69* gene product, Velasco et al. (2006) argued that the BRAM1 cDNA is in fact an artificial chimeric product between *Anks1* and *Bs69* sequences that happened from a recombination event during the cDNA library construction. Zebrafish Bram1 cDNA, isolated from a cDNA library by RACE technique, is merely a C-terminal part of the full length Bs69 cDNA, as the proposed zebrafish *bram1* gene encodes a peptide lacks the featured MLLEPPSPVPW sequences like its mammalian counterparts. Moreover, we failed to amplify any Bram1-like cDNAs from our zebrafish cDNA libraries. Thus, we think that no Bram1 type exists in zebrafish. In this context, we are the first to dissect the developmental function of Bs69 using zebrafish as a model. In this work, by loss of function and overexpression assays, we demonstrated that zebrafish Bs69 is a negative regulator of Bmp signaling by physically interacting with Bmpr1a. The Mynd domain of Bs69 is indispensible for this function as it mediates Bs69 binding to the kinase domain of Bmpr1a.

The majority of studies so far proposed that BS69 function as co-factor for transcriptional or chromatin regulation in the nucleus (Hateboer et al., 1995; Guo et al., 2014; Wen et al., 2014). In recent years, however, a growing body of work have demonstrated that BS69 has important roles in the regulation of signaling pathways in the cytoplasmic membrane. For instances, BS69 was shown to interact with multiple *trans*-membrane proteins, including LMP1, LTβR, and BMPR1A (Kurozumi et al., 1998; Liu et al., 2011; this study). BS69 is also constitutively co-localized in the membrane lipid rafts in mammalian cells (Wan et al., 2006). Lipid rafts were proposed to function in membrane protein sorting and in the formation of signaling complexes, as well as in endocytic trafficking (Hartung et al., 2006). It is possible that Bs69 is localized in membrane lipid rafts in zebrafish cells and may be involved in the endocytosis of Bmp receptors or the formation of ligand-receptor complex. In the future, it will be interesting to look into this possibility.

### Mga Interacts With Bs69 to Regulate Bmp Signaling

MAX giant associated protein is a transcriptional factor containing both T-box and bHLH domains, and was proposed to regulate the expression of both Max-network or T-box family genes (Hurlin et al., 1999). The Myc family of transcriptional factors are known for their role in the control of cell cycle progression, cellular growth and proliferation (Gallant, 2006). The T-box family of transcriptional factors play key role in mesendoderm formation in vertebrate embryogenesis (Papaioannou, 2014). Surprisingly, our *mga* mutant zebrafish are viable, and can grow up to adult without obvious morphological defects, except the loss or reduction of ventral tailfin. This fact indicates that Mga is not critically required for cell cycle progression and cell proliferation in zebrafish, and that Mga has limited function as the member of the T-box family of transcriptional factors. Alternatively, there are certain compensation mechanisms to allow normal zebrafish development in the absence of Mga. In this context, our work revealed a different requirement of MGA for zebrafish and mice embryonic development.

Our previous work suggested that Mga:Max could transcriptionally regulate bmp2b expression by binding to its promotor or enhancer in YSL in zebrafish. Now, we show that Mga localized in the cytoplasm directly interacts with and antagonizes Bs69 to modulate Bmpr1a-mediated Bmp signaling. This surprising role of Mga protein is executed in the cytoplasm, does not require dimerization with Max, and is independent of its transcriptional or chromatin remodeling activities. This is consistent with the observation that Mga is predominantly localized in the cytoplasm throughout zebrafish early embryogenesis. Indeed, mouse MGA is also predominantly localized in the cytoplasm (data not shown), suggesting that similar mechanism for the regulation of Bmp signaling occurs in mammals. We found that zebrafish Mga physically associates with Bs69 in the physiological conditions and this interaction is mediated by the PXLXP motif of Mga and the Mynd domain of Bs69. Mga-Cter binding to Bs69 disrupts the Bs69–Bmpr1a association which allows proper Bmp signaling to be maintained. By applying mga morpholino, we depleted Mga in *bs69* mutant background. And we found that embryos depleted of both proteins had a similar DV patterning phenotype to bs69 mutant animals. This data further supported our hypotheses that Mga regulates Bmp signaling through Bs69–Bmpr1a axis.

Our previous work showed that Mga-Cter also binds to Smad4 and Smad1, two core components of Bmp signaling pathway (Sun et al., 2014). It is possible that Mga, Bs69, and Smad4 could form a triplex or Bs69 competes with Smad4 for binding to Mga. In any case, Mga binding to Bs69 could even enhance Bmp signaling by simultaneously antagonizing Bs69 and promoting the formation of Smad4: pSmad1/5/8 complex at the cytoplasmic membrane via releasing or bringing Mga bound Smad4 or Smad1 to Bmpr1a. This may explain why expressing both Bs69 and Mga had significantly stronger effect on Bmp activity than expressing Mga only (**Figures 5D, 6F**). Altogether, these data indicate that Mga functions to control Bmp signaling pathways in different cellular compartments, at different levels and through different mechanisms. To our knowledge, this is the first report showing that certain member of the Myc or T-box family of transcriptional factors regulates signaling pathways in the cytoplasm. Nevertheless, *mga* mutant embryos at 1 dpf displayed mild DV patterning defects, suggesting that Mga acts to fine-tune Bmp signaling in zebrafish.

In addition to the regulation of Bmp signaling, both Bs69 and Mga are known chromatin readers and remodelers, raising an interesting question whether Mga and Bs69 could link Bmp signaling to chromatin structure regulation or transcriptional elongation (Velasco et al., 2006; Guo et al., 2014). Answering this question will help us to understand how signal transduction pathways directly communicate with chromatin to change the epigenetic landscape or gene expression. With *mga* and *bs69* mutant zebrafish in hand, this question is under investigation in our lab.

## Ethics Statement

This study was carried out in accordance with the recommendations of ethical approval had been obtained from Animal ethical committee of Institute of hydrobiology for the approval of animal experiments. The protocol was approved by the “Animal Ethical Committee.”

## Author Contributions

YS and SD designed the experiments. XS, JC, YZ, and MM performed the experiments. YS wrote the manuscript.

## Conflict of Interest Statement

The authors declare that the research was conducted in the absence of any commercial or financial relationships that could be construed as a potential conflict of interest.

## Footnotes

1 http://zifit.partners.org/ZiFiT/

## Supplementary Material

FIGURE S1 **(A)** The phenotypes of 1 dpf wild type embryos treated with 0.05 μM DMSO, or 0.05 μM dorsomorphin or 0.05 μM LDN193189 starting at one-cell stage, or injected with 50 pg *dnBmpr1a* mRNA at one-cell-stage. **(B)** 50 pg *caBmpr1a* mRNA rescued the loss of ventral tail fin phenotype of *mga* mutant embryos. Shown are representative embryos at 2 dpf. **(C)** WISH for 72 hpf embryos using *mga, bs69*, and *bmpr1aa* probes. *mga* and *bs69* probes are DIG-labeled, whereas *bmpr1aa* probe is fluorescein-labeled.

FIGURE S2 **(A)** The co-localization of Mga and HA-Bs69-Mynd in 7 hpf embryos. **(B)** The co-localization of zebrafish Mga and HA-Bs69 in 293T cells. **(C)** The co-localization of zebrafish Mga and HA-Bs69-Mynd in 293T cells.

FIGURE S3 **(A)** Dorsalized phenotypes of HA-Bs69-Mynd overexpressing embryos at 2 dpf. C1-4 classification according to DV patterning index. **(B)** Western blot analysis of lysates from 8 hpf HA-Bs69-Mynd overexpressing or control embryos. **(C)** Tail region of 2 dpf *bs69* mutant embryos injected at one-cell-stage with 50 pg mRNAs encoding HA-Bs69-Mynd or β-Gal.

FIGURE S4 **(A)** DV patterning phenotypes of *bs69^-/-^* mutant embryos at 1 dpf injected with 4 ng mgamisMO or mgaMO. V1-2 classification according to DV patterning index. **(B)** Quantification of **(A)** based on three independent experiments.

FIGURE S5 **(A)** The cardiac laterality defects of *mga* mutant or Bs69 overexpressing embryos at 1 dpf revealed by WISH using *spaw, lefty2*, and *cmlc2* probes. **(B)** Percentage of embryos that exhibited cardiac laterality defects. L, left; M, middle; R, right. **(C)** 50 pg mouse *mga* mRNA partially rescued the cardiac laterality defects of *mga* mutants at 1 dpf; 50 pg *caBmpr1a* mRNA partially rescued the cardiac laterality defects of Bs69 overexpressing embryos at 1 dpf.

## References

[B1] Ansieau S. Leutz A. (2002). The conserved Mynd domain of BS69 binds cellular and oncoviral proteins through a common PXLXP motif. *J. Biol. Chem.* 277 4906–4910. 10.1074/jbc.M110078200 11733528

[B2] Chen J. Cui X. J. Jia S. T. Luo D. J. Cao M. X. Zhang Y. S. (2016). Disruption of dmc1 produces abnormal sperm in medaka (*Oryzias latipes*). *Sci. Rep.* 6:30912. 10.1038/srep30912 27480068 PMC4969596

[B3] Chung P. J. Chang Y. S. Liang C. L. Meng C. L. (2002). BRAM1 functions by the bone morphogenetic protein receptor IA-binding protein, negative regulation of epstein-barr virus latent membrane protein 1-mediated. *Biol. Chem.* 277 39850–39857. 10.1074/jbc.M206736200 12181323

[B4] De Paoli L. Cerri M. Monti S. Rasi S. Spina V. Bruscaggin A. (2013). MGA, a suppressor of MYC, is recurrently inactivated in high risk chronic lymphocytic leukemia. *Leukemia Lymphoma* 54 1087–1090. 10.3109/10428194.2012.723706 23039309

[B5] Endoh M. Endo T. Shinga J. Hayashi K. Farcas A. Ma K. W. (2017). PCGF6-PRC1 suppresses premature differentiation of mouse embryonic stem cells by regulating germ cell-related genes. *eLife* 6:e21064. 10.7554/eLife.21064 28304275 PMC5375644

[B6] Gallant P. (2006). Myc/Max/Mad in invertebrates: the evolution of the max network. *Curr. Top. Microbiol. Immunol.* 302 235–253. 16620031 10.1007/3-540-32952-8_9

[B7] Gao Z. H. Zhang J. Bonasio R. Strino F. Sawai A. Parisi F. (2012). PCGF homologs, CBX proteins, and RYBP define functionally distinct PRC1 family complexes. *Mol. Cell* 45 344–356. 10.1016/j.molcel.2012.01.002 22325352 PMC3293217

[B8] Guo R. Zheng L. J. Park J. W. Lv R. Chen H. Jiao F. F. (2014). BS69/ZMYND11 reads and connects histone H3.3 lysine 36 trimethylation-decorated chromatin to regulated pre-mRNA processing. *Mol. Cell* 56 298–310. 10.1016/j.molcel.2014.08.022 25263594 PMC4363072

[B9] Harter M. R. Liu C. D. Shen C. L. Gonzalez-Hurtado E. Zhang Z. M. Xu M. (2016). BS69/ZMYND11 C-terminal domains bind and inhibit EBNA2. *PLoS Pathog.* 12:e1005414. 10.1371/journal.ppat.1005414 26845565 PMC4742278

[B10] Hartung A. Bitton-Worms K. Rechtman M. M. Wenzel V. Boergermann J. H. Hassel S. (2006). Different routes of bone morphogenic protein (BMP) receptor endocytosis influence BMP signaling. *Mol. Cell. Biol.* 26 7791–7805. 10.1128/MCB.00022-06 16923969 PMC1636853

[B11] Hateboer G. Gennissen A. Ramos Y. F. Kerkhoven R. M. Sonntag-Buckl V. Stunnenberg H. G. (1995). BS69, a novel adenovirus ElA-associated protein that inhibits EIA transactivation. *EMBO J.* 14 3159–3169. 10.1002/j.1460-2075.1995.tb07318.x 7621829 PMC394377

[B12] Hurlin P. J. Steingrımsson E. Copeland N. G. Jenkins N. A. Eisenman R. N. (1999). Mga, a dual-specificity transcription factor that interacts with Max and contains a T-domain DNA-binding motif. *EMBO J.* 18 7019–7028. 10.1093/emboj/18.24.7019 10601024 PMC1171765

[B13] Ikeda O. Sekine Y. Mizushima A. Oritani K. Yasui T. Fujimuro M. (2009). BS69 negatively regulates the canonical NF-jB activation induced by epstein–barr virus-derived LMP1. *FEBS Lett.* 583 1567–1574. 10.1016/j.febslet.2009.04.022 19379743

[B14] Jo Y. S. Kim M. S. Yoo N. J. Lee S. H. (2016). Somatic mutation of a candidate tumour suppressor MGA gene and its mutational heterogeneity in colorectal cancers. *Pathology* 48 525–527. 10.1016/j.pathol.2016.04.010 27306572

[B15] Katagiri T. Watabe T. (2016). Bone Morphogenetic Proteins. *Cold Spring Harbor Perspect. Biol.* 22 233–241. 10.1101/cshperspect.a021899 27252362 PMC4888821

[B16] Kishimoto Y. Lee K. H. Zon L. Hammerschmidt M. Schulte-Merker S. (1997). The molecular nature of zebrafish swirl: BMP2 function is essential during early dorsoventral patterning. *Development* 124 4457–4466. 9409664 10.1242/dev.124.22.4457

[B17] Kurozumi K. Nishita M. Yamaguchi K. Fujita T. Ueno N. Shibuya H. (1998). BRAM1, a BMP receptor-associated molecule involved in BMP signaling. *Genes Cells* 3 257–264. 10.1046/j.1365-2443.1998.00186.x 9663660

[B18] la Cour T. Kiemer L. Mølgaard A. Gupta R. Skriver K. Brunak S. (2004). Analysis and prediction of leucine-rich nuclear export signals. *Protein Eng. Des. Sel.* 17 527–536. 10.1093/protein/gzh062 15314210

[B19] Liu H. P. Chung P. J. Liang C. L. Chang Y. S. (2011). The MYND domain-containing protein BRAM1 inhibits lymphotoxin beta receptor-mediated signaling through affecting receptor oligomerization. *Cell. Signal.* 23 80–88. 10.1016/j.cellsig.2010.08.006 20732415

[B20] Liu X. Chen Z. Xu C. X. Leng X. Q. Cao H. Ouyang G. (2015). Repression of hypoxia-inducible factor α signaling by set7-mediated methylation. *Nucleic Acids Res.* 43 5081–5098. 10.1093/nar/gkv379 25897119 PMC4446437

[B21] Morita K. Shimizu M. Shibuya H. Ueno N. (2001). A DAF-1-binding protein BRA-1 is a negative regulator of DAF-7 TGF-b signaling. *PNAS* 98 6284–6288. 10.1073/pnas.111409798 11353865 PMC33460

[B22] Nguyen B. Pogoutse A. Provart N. Moses A. (2009). NLStradamus: a simple Hidden Markov Model for nuclear localization signal prediction. *BMC Bioinformatics* 10:202. 10.1186/1471-2105-10-202 19563654 PMC2711084

[B23] Nikaido M. Tada M. Takeda H. Kuroiwa A. Ueno N. (1999). In vivo analysis using variants of zebrafish BMPR-IA: range of action and involvement of BMP in ectoderm patterning. *Development* 126 181–190. 9834197 10.1242/dev.126.1.181

[B24] Ogawa H. Ishiguro K. Gaubatz S. Livingston D. M. Nakatani Y. (2002). A complex with chromatin modifiers that occupies E2F- and Myc-responsive genes in G0 cells. *Science* 296 1132–1136. 10.1126/science.1069861 12004135

[B25] Papaioannou V. E. (2014). The T-box gene family: emerging roles in development, stem cells and cancer. *Development* 141 3819–3833. 10.1242/dev.104471 25294936 PMC4197708

[B26] Pyati U. J. Webb A. E. Kimelman D. (2005). Transgenic zebrafish reveal stage-specific roles for Bmp signaling in ventral and posterior mesoderm development. *Development.* 132 2333–2343. 10.1242/dev.01806 15829520

[B27] Rikin A. Evans T. (2010). The tbx/bHLH transcription factor Mga regulates gata4 and organogenesis. *Dev. Dyn.* 239 535–547. 10.1002/dvdy.22197 20044811 PMC3613857

[B28] Schumacher J. A. Hashiguchi M. Nguyen V. H. Mullins M. C. (2011). An intermediate level of BMP signaling directly specifies cranial neural crest progenitor cells in zebrafish. *PLoS One* 6:e27403. 10.1371/journal.pone.0027403 22102893 PMC3216922

[B29] Smith K. A. Noel E. Thurlings I. Rehmann H. Chocron S. Bakkers J. (2011). Bmp and Nodal independently regulate lefty1 expression to maintain unilateral Nodal activity during left-right axis specification in zebrafish. *PLoS Genet.* 7:e1002289. 10.1371/journal.pgen.1002289 21980297 PMC3183088

[B30] Sun Y. H. Tseng W. C. Ball R. Dougan S. (2014). Extra-embryonic signals under the control of Mga, Max and Smad4 are required for dorsoventral patterning. *Dev. Cell* 28 322–334. 10.1016/j.devcel.2014.01.003 24525188

[B31] Sun Y. H. Wloga D. Dougan S. (2011). Embryological manipulations in zebrafish. *Methods Mol. Biol.* 770 139–184. 10.1007/978-1-61779-210-6_6 21805264

[B32] Suzuki A. Hirasaki M. Hishida T. Wu J. Okamura D. (2017). Loss of MAX results in meiotic entry in mouse embryonic and germline stem cells. *Nat. Commun.* 7:11056. 10.1038/ncomms11056 27025988 PMC4820925

[B33] The Cancer Genome Atlas Research Network (2014). Comprehensive molecular profiling of lung adenocarcinoma. *Nature* 511 543–550. 10.1038/nature13385 25079552 PMC4231481

[B34] Velasco G. Grkovic S. Ansieau S. (2006). New insights into BS69 functions. *J. Biol. Chem.* 281 16546–16550. 10.1074/jbc.M600573200 16565076

[B35] Wan J. Zhang W. Wu L. Bai T. Zhang M. Lo K. W. (2006). BS69, a specific adaptor in the latent membrane protein 1-mediated c-Jun N-terminal kinase pathway. *Mol. Cell. Biol.* 26 448–456. 10.1128/MCB.26.2.448-456.2006 16382137 PMC1346911

[B36] Wang R. N. Green J. Wang Z. L. Deng Y. L. Qiao M. Peabody M. (2014). Bone morphogenetic protein (BMP) signaling in development and human diseases. *Genes Dis.* 1 87–105. 10.1016/j.gendis.2014.07.005 25401122 PMC4232216

[B37] Washkowitz A. J. Schall C. Zhang K. Wurst W. Floss T. Mager J. (2015). Papaioannou. Mga is essential for the survival of pluripotent cells during peri-implantation development. *Development* 142 31–40. 10.1242/dev.111104 25516968 PMC4299147

[B38] Wen H. Li Y. Y. Xi Y. X. Jiang S. M. Stratton S. Peng D. (2014). ZMYND11 links histoneH3.3K36me3 to transcription elongation and tumour suppression. *Nature* 508 263–268. 10.1038/nature13045 24590075 PMC4142212

[B39] Wu K. M. Huang C. J. Hwang S. P. Chang Y. S. (2006). Molecular cloning, expression and characterization of the zebrafish bram1 gene, a BMP receptor-associated molecule. *J. Biomed. Sci.* 13 345–355. 10.1007/s11373-005-9066-2 16456708

