# Supplementary figures and images for: Mga Modulates Bmpr1a Activity by Antagonizing Bs69 in Zebrafish
Source: Front Cell Dev Biol. 2018 Sep 28;6:126. doi: 10.3389/fcell.2018.00126 (PMC6172302; doi:10.3389/fcell.2018.00126)

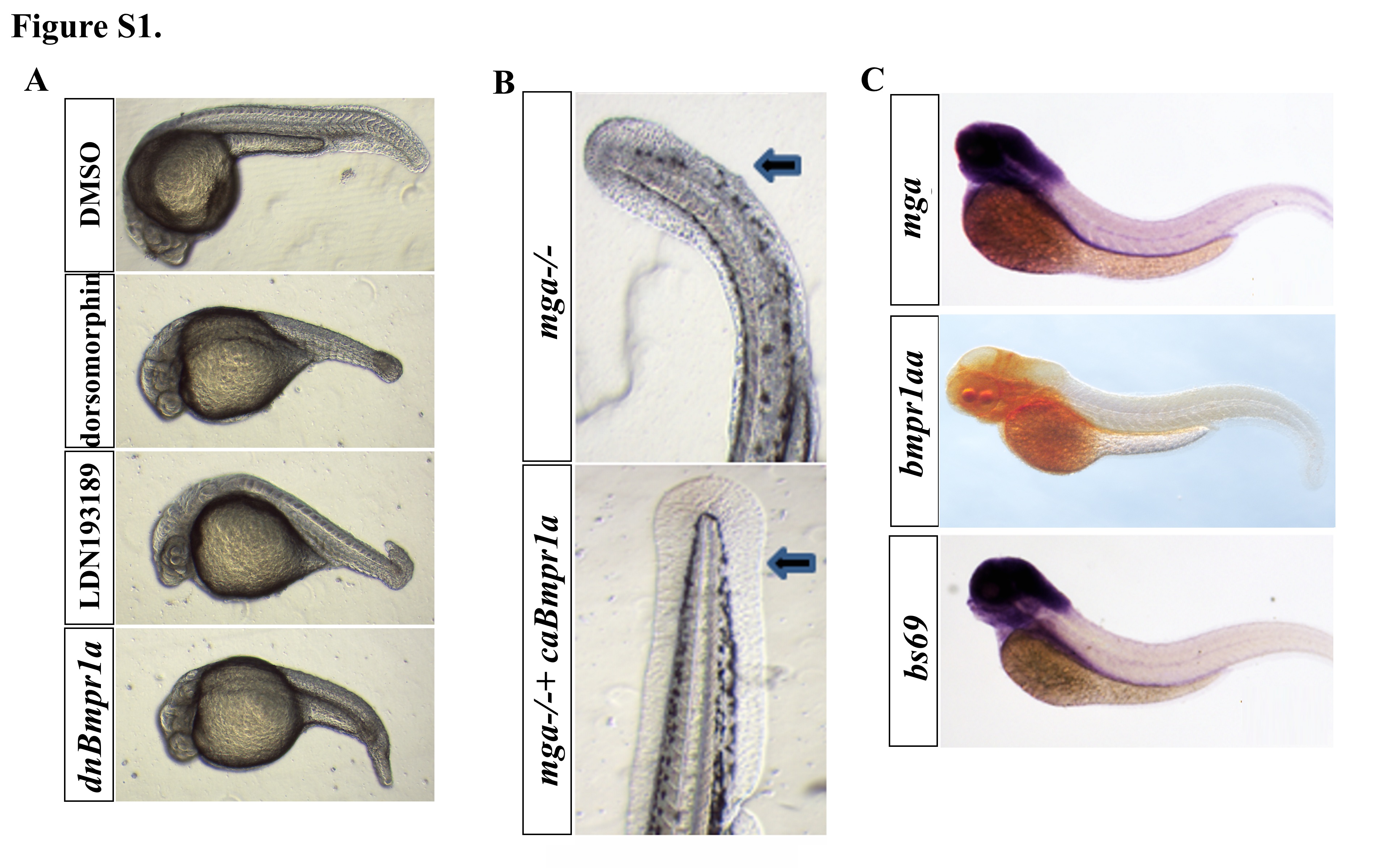

Supplement: FIGURE S1 — (A) The phenotypes of 1 dpf wild type embryos treated with 0.05 μM DMSO, or 0.05 μM dorsomorphin or 0.05 μM LDN193189 starting at one-cell stage, or injected with 50 pg dnBmpr1a mRNA at one-cell-stage. (B) 50 pg caBmpr1a mRNA rescued the loss of ventral tail fin phenotype of mga mutant embryos. Shown are representative embryos at 2 dpf. (C) WISH for 72 hpf embryos using mga, bs69, and bmpr1aa probes. mga and bs69 probes are DIG-labeled, whereas bmpr1aa probe is fluorescein-labeled. [file Image_1.JPEG]

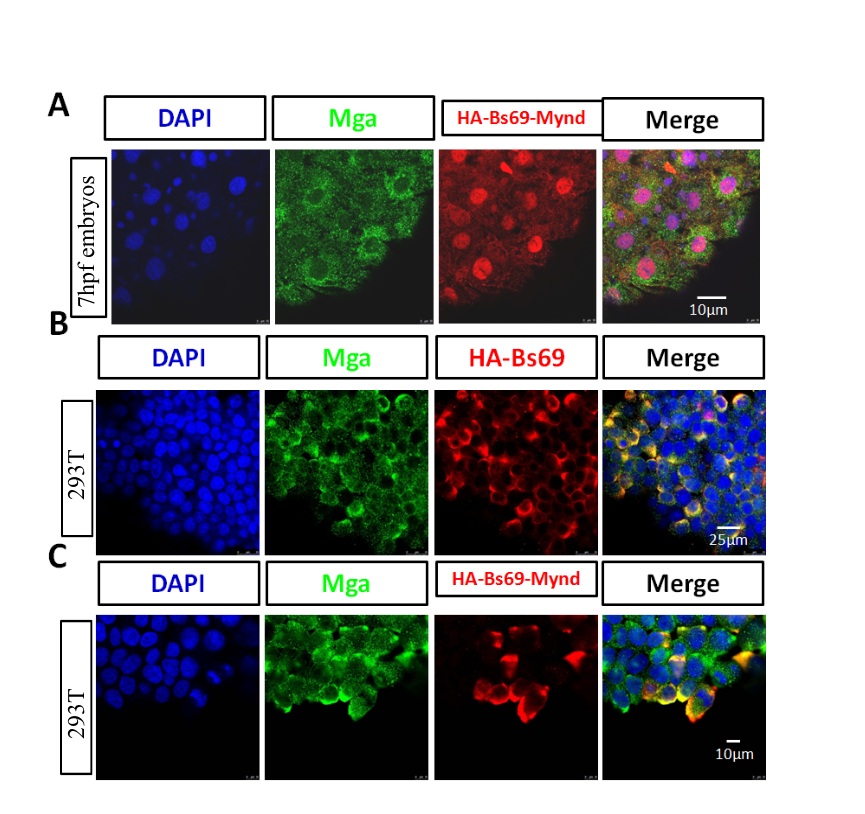

Supplement: FIGURE S2 — (A) The co-localization of Mga and HA-Bs69-Mynd in 7 hpf embryos. (B) The co-localization of zebrafish Mga and HA-Bs69 in 293T cells. (C) The co-localization of zebrafish Mga and HA-Bs69-Mynd in 293T cells. [file Image_2.JPEG]

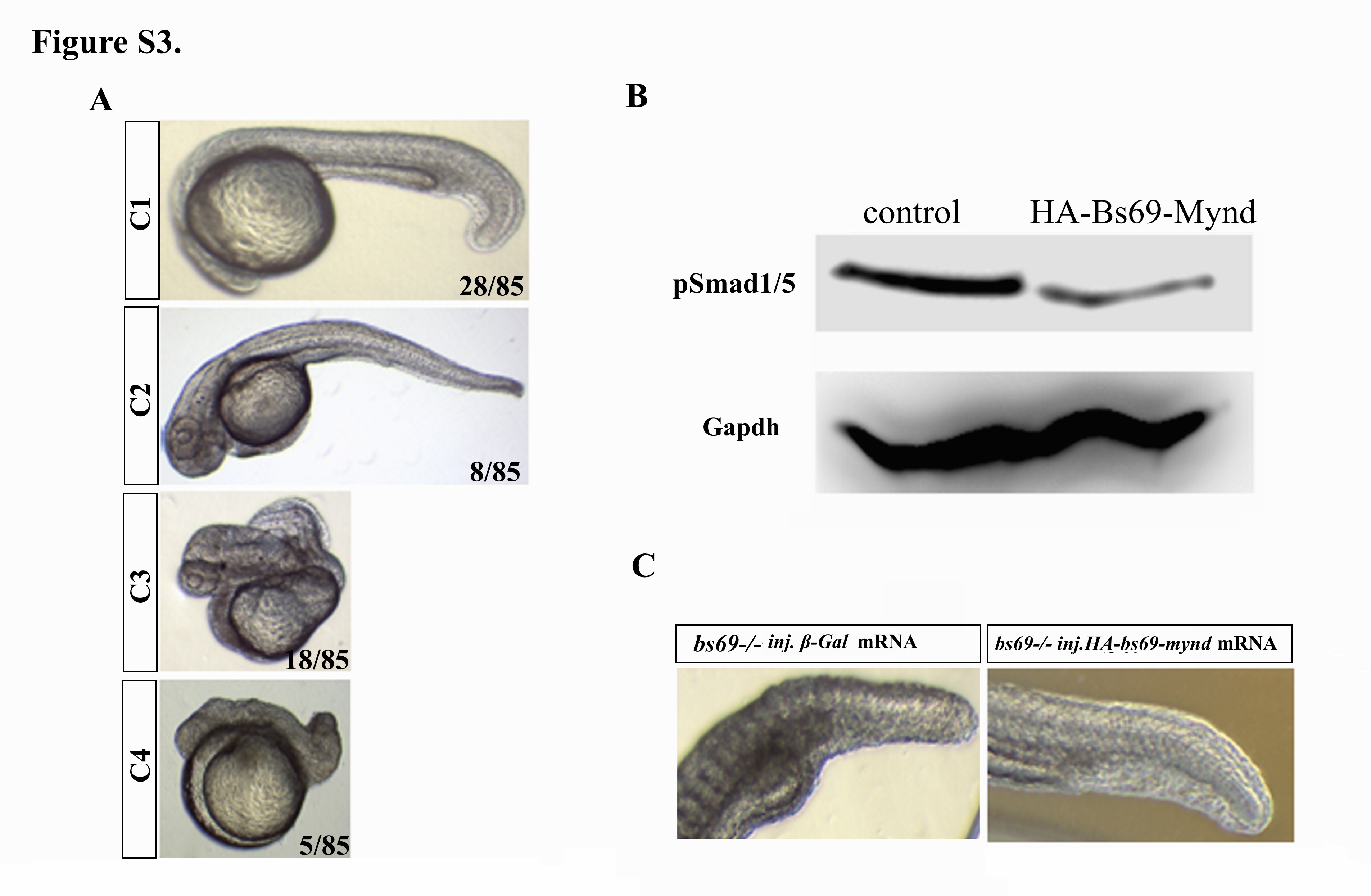

Supplement: FIGURE S3 — (A) Dorsalized phenotypes of HA-Bs69-Mynd overexpressing embryos at 2 dpf. C1-4 classification according to DV patterning index. (B) Western blot analysis of lysates from 8 hpf HA-Bs69-Mynd overexpressing or control embryos. (C) Tail region of 2 dpf bs69 mutant embryos injected at one-cell-stage with 50 pg mRNAs encoding HA-Bs69-Mynd or β-Gal. [file Image_3.JPEG]

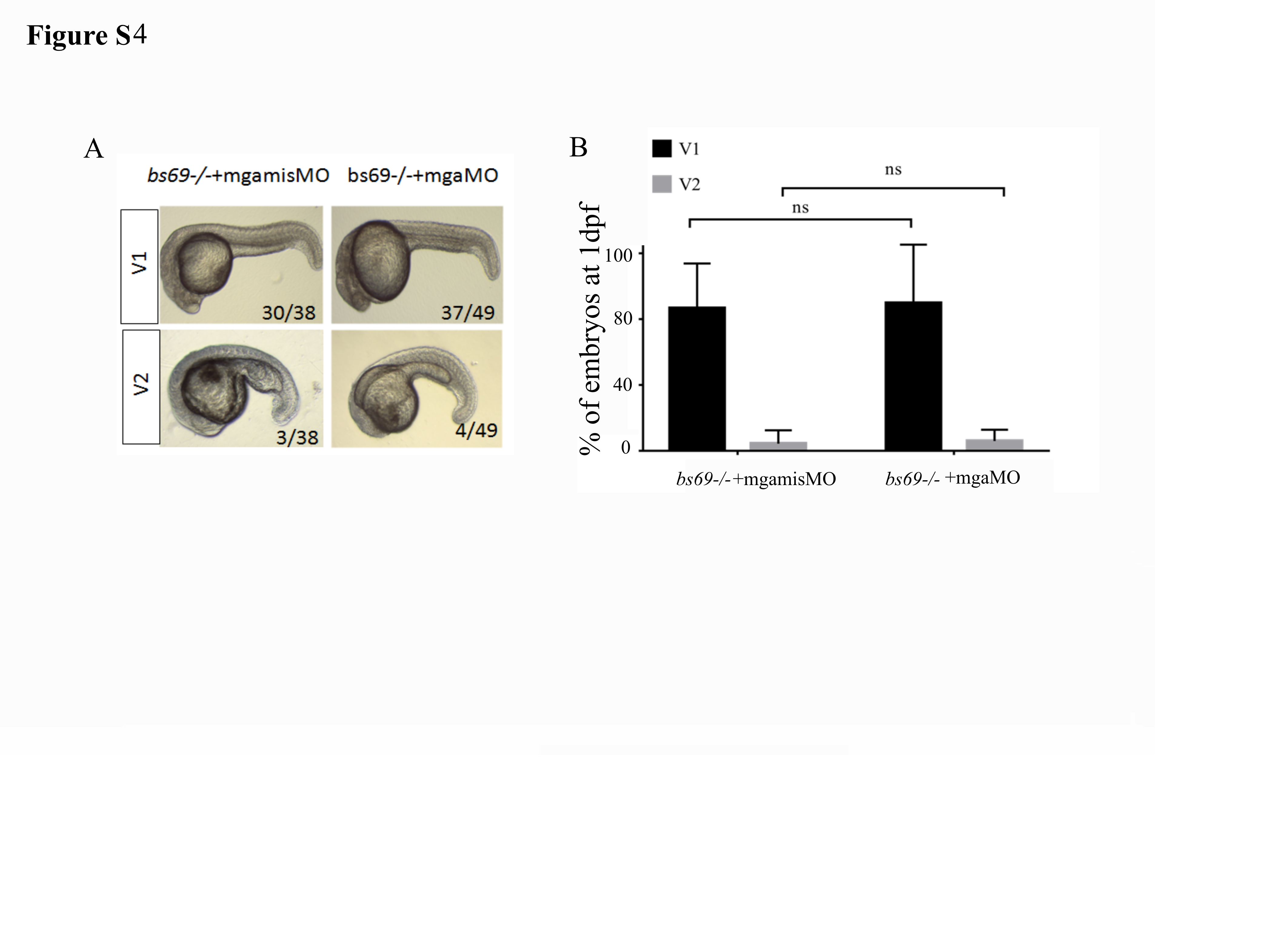

Supplement: FIGURE S4 — (A) DV patterning phenotypes of bs69-/- mutant embryos at 1 dpf injected with 4 ng mgamisMO or mgaMO. V1-2 classification according to DV patterning index. (B) Quantification of (A) based on three independent experiments. [file Image_4.TIF]

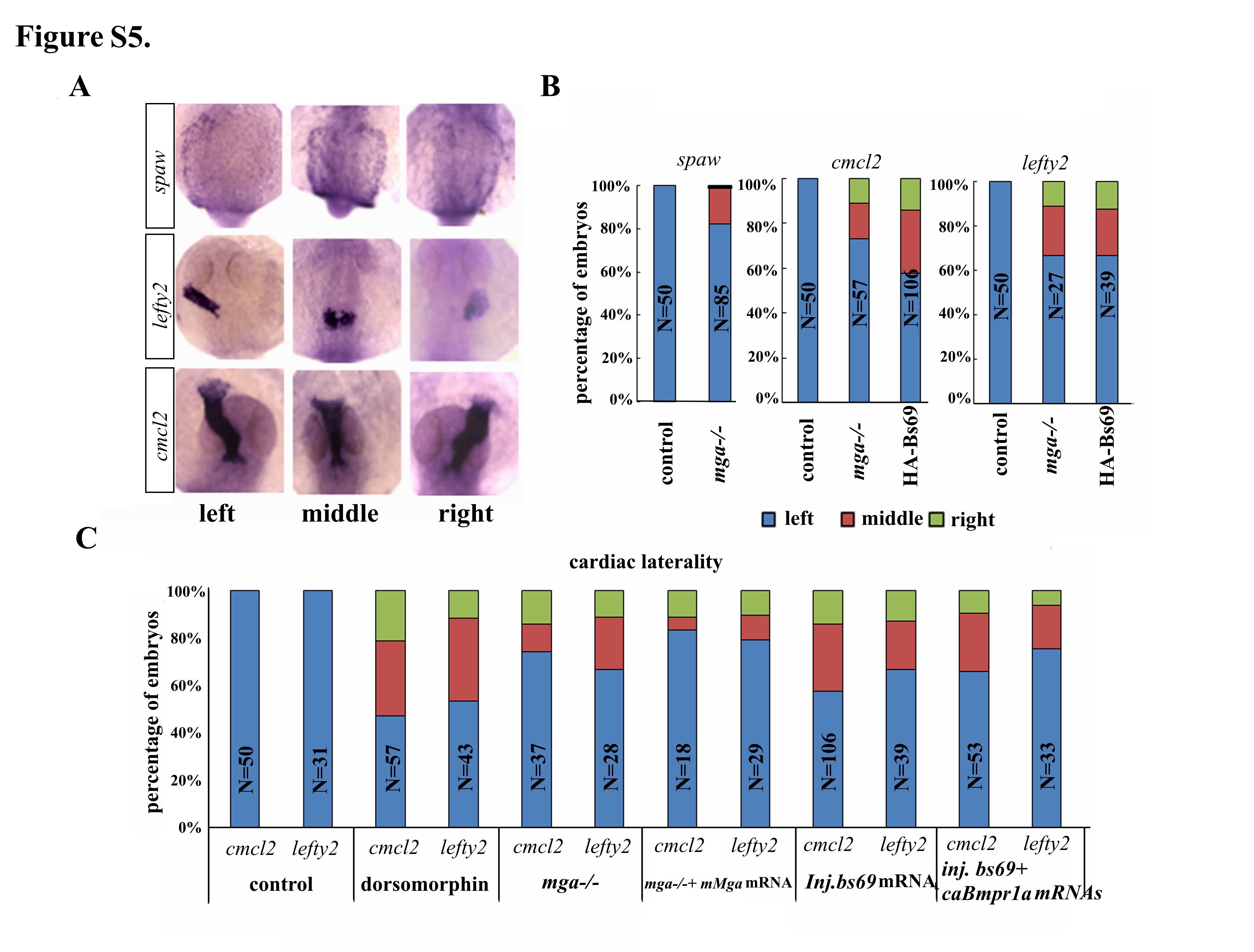

Supplement: FIGURE S5 — (A) The cardiac laterality defects of mga mutant or Bs69 overexpressing embryos at 1 dpf revealed by WISH using spaw, lefty2, and cmlc2 probes. (B) Percentage of embryos that exhibited cardiac laterality defects. L, left; M, middle; R, right. (C) 50 pg mouse mga mRNA partially rescued the cardiac laterality defects of mga mutants at 1 dpf; 50 pg caBmpr1a mRNA partially rescued the cardiac laterality defects of Bs69 overexpressing embryos at 1 dpf. [file Image_5.JPEG]
